# Methodology of community-based venous blood specimen collection for the harmonized diagnostic assessment of dementia for the Longitudinal Aging Study in India (LASI-DAD): Wave 2

**DOI:** 10.1371/journal.pone.0326917

**Published:** 2025-07-31

**Authors:** Anushikha Dhankhar, Pranali Khobragade, Joyita Banerjee, Geeta Chopra, Steffi Jacob, A.B. Dey, Jinkook Lee, Eileen Crimmins, Bharat Thyagarajan, Sharmistha Dey, Peifeng Hu

**Affiliations:** 1 Center for Economic and Social Research, University of Southern California, Los Angeles, California, United States of America; 2 Metropolis Healthcare Ltd., Delhi, India; 3 Mobile Creches, Delhi, India; 4 Venu Geriatric Institute, Venu Eye Hospital and Research Centre, Delhi, India; 5 Department of Economics, University of Southern California, Los Angeles, California, United States of America; 6 Davis School of Gerontology, University of Southern California, Los Angeles, California, United States of America; 7 Department of Laboratory Medicine and Pathology, Division of Molecular Pathology and Genomics, University of Minnesota Twin Cities, Minnesota, United States of America; 8 Department of Biophysics, All India Institute of Medical Sciences, Delhi, India; 9 Division of Geriatrics, David Geffen School of Medicine, University of California Los Angeles, Los Angeles, California, United States of America; Rutgers University New Brunswick, UNITED STATES OF AMERICA

## Abstract

The prevalence of dementia is on the rise, with 60% of dementia cases existing in low- and middle-income countries. In India, the prevalence was reported to be 7.4%. Since the pathophysiology of dementia is multifactorial, the Harmonized Longitudinal Aging Study in India for the Diagnostic Assessment of Dementia (LASI-DAD) collected data to capture multiple domains, including venous blood specimens (VBS). VBS collection and assays help ascertain the overall health status of an individual, understand disease pathogenesis, and diagnose diseases. In community settings, blood assays also help identify disease trends. However, community VBS collections can often be challenging. Sample quality can be impaired due to individual, environmental, geographical, and pre-analytical processing factors. Therefore, standardization of the process is imperative to ensure biomarker data of high accuracy. LASI-DAD developed a systematic sample collection, shipment, processing, and storage protocol. Multiple checkpoints were in place to monitor sample quality in real time. A phlebotomist was trained from each participating state for specimen collection. All samples were centrally tested for analytes. The overall response rate for blood collection was 71.5%. We collected 17 mL of VBS from 3,252 respondents, who consented to participate. Blood samples were tested for routine analytes, and those specific to Alzheimer’s Disease (AD) and AD-related dementias (ADRD). Data was reviewed fortnightly. The median cold chain temperature was 6.2°C and hemolysis was seen in 6.7% of the samples. LASI-DAD standardized and implemented VBS collection while overcoming the challenges faced due to India’s diverse socio-demographic, geographical, and environmental conditions. This methodology can serve as a robust tool for VBS handling and ensuring high sample quality for future community-based studies.

## Introduction

Dementia usually presents in late life as a progressive deterioration of cognitive skills, memory, and reasoning abilities, to the extent of disrupting the person’s daily activities [1]. This syndrome may be caused by multiple diseases of the brain, the most common one being Alzheimer’s disease (AD) [2]. At present, low- and middle-income countries contribute to approximately 60% of all dementia cases [3]. In India, the prevalence of dementia was reported to be 7.4% (about 8.8 million) among adults aged 60 years and above [4]. Aging has been identified as posing the greatest risk of developing dementia [5]. Over the last few decades, India has observed an increasing life expectancy [6], a progressively aging population, and is recorded to have the largest population in the world at present [7]. Owing to these factors, India is expected to witness an exponential rise in the number of people with dementia in the coming years [4]. Multiple underlying risk factors are indicated to play a role in the pathogenesis of dementia. In addition to aging [5] and neurodegenerative diseases [8], cardiovascular risk factors such as diabetes and high blood pressure [9–12], an individual’s genetic predisposition [13], and unfavorable lifestyle factors [10,13] may also increase the likelihood of cerebrovascular changes in the brain that can lead to dementia. The Harmonized Diagnostic Assessment of Dementia for the Longitudinal Aging Study in India (LASI-DAD) addressed the pressing need for robust population-based research to diagnose dementia and its risk factors in India [14]. The battery of diagnostic tools used in the study includes cognitive tests, neuroimaging, physical and environmental markers, nutrition, and venous blood-based assays. The detailed protocol of the study has been published elsewhere [15]. This paper primarily focuses on community-based venous blood specimen (VBS) collection in the LASI-DAD study.

VBS collection is done to assess an individual’s health status, and diagnose, monitor, manage, and treat diseases [16,17]. When conducted on a community level, VBS analysis helps to reflect objectively upon the health outcomes and the determinants of health in a population. It also aids in identifying disease trends, determining the prevalence or incidence of risk factors, and high-risk or vulnerable population groups for the disease under study [18]. However, it emanates significant challenges [19] during mass specimen collection, sample transportation under appropriate temperatures, and timely pre-analytical processing, especially in developing countries like India with diverse climatic, environmental, and geographical conditions and disparate resource availability. These factors may tremendously impact sample quality, which, in turn, has direct analytical and diagnostic implications.

Previously, population-based studies in India have primarily focused on collecting dried blood spots (DBS) for screening and diagnostic purposes [20–22]. DBS collection is less painful, more feasible for large-scale specimen collection, and comparatively inexpensive. However, it allows the testing of only a limited number of analytes, and the variability in biomarker measurement using DBS is substantially higher than that obtained from venous blood. Therefore, the LASI-DAD study [14] assayed venous blood samples collected from a nationally representative population to objectively measure and study biomarkers and risk factors for dementia in India [15,23]. Previous literature suggests that non-conformities in pre-analytical sample handling are major causes of systematic errors and inaccuracy in assay results [18]. For instance, the concentration of some analytes, such as creatinine, hemoglobin, folate, and markers of AD and AD-related dementias (ADRD), is prone to changing with a delay in processing the blood sample or a rise in cold-chain temperature [24,25]. Therefore, having rigorous collection and processing protocols is crucial to maintaining the quality of blood samples at each step to ensure the accuracy of the assay results [25], especially in a nationally representative study. In this paper, we discuss the meticulous methodology adopted for the collection, shipment, processing, and storage of high-quality VBS for wave 2 of the LASI-DAD study, and provide indicators of sample quality.

### Participants and setting

Blood samples were collected in a community setting from study participants aged 60 years and above. Our study participants included both males and females from rural and urban areas of 22 states and union territories of India, thus, representing the national sample population.

## Materials and methods

VBS collection was carried out in all the participating states and union territories in India in association with Metropolis Healthcare Limited [26], a third-party diagnostic and test service provider laboratory. Metropolis Healthcare has a wide operational network across India. Metropolis Healthcare Ltd, Delhi, the central sample processing unit, is accredited by the National Accreditation Board for Testing and Calibration Laboratories (NABL), where routine sample testing was performed. Ethics approval was obtained from the Institute Ethics Committee of All India Institute of Medical Sciences (AIIMS), New Delhi, all collaborating medical institutions, and the University of Southern California (UP-15–00684). The Health Ministry Screening Committee of the Indian Council of Medical Research provided administrative clearance to the LASI-DAD research study (2202–16741/F1). A panel of experts from the University of Southern California, AIIMS New Delhi, University of California Los Angeles, University of Minnesota, and Metropolis Healthcare Limited Inc. India, developed, reviewed, and revised the protocol and standard operating procedure (SOP) as deemed necessary during the course of the study. A team of field investigators, a supervisor, and a phlebotomist assigned by Metropolis were trained to carry out and assist in VBS collection in each state. Earlier in the study, we trained multiple phlebotomists from local laboratories closer to the respondents’ location. However, subsequently, we adopted a protocol where one phlebotomist was hired per state to standardize the procedure and minimize bias [27]. Emphasis was given to respondent safety, confidentiality, and SOP for sample collection, processing, packaging, shipment, quality check, and reporting unfavorable events at any step of the process.

### Field operations and venous blood specimen collection

Field investigators conducted the LASI-DAD survey interviews and accompanied the phlebotomist for blood draws in each state. The study participants, the phlebotomist, and the Metropolis Healthcare logistics team were notified of the blood draw schedule 48 hours in advance to allow for adequate preparation time. Before blood collection began, a written informed consent form was obtained from the respondents in the presence of a family member to explain the procedure. For cognitively impaired participants, consent was obtained from a legal representative authorized to sign on their behalf. Informed consent was collected in the participant’s language, and consent documents were translated into 12 Indian languages. Educating and informing participants about VBS collection helped respondents understand the rationale for the request, clarified misconceptions, built rapport with them, and encouraged participant cooperation, especially among individuals and families with negative perceptions about blood sample collection [28]. Those who agreed to participate signed the consent form or provided a thumbprint. A total of 17 mL of fasting blood sample in two serum separation tubes (SSTs) of 3.5 mL each (tubes A and B), 2 mL (tube C) and 3 mL (tube D) EDTA tubes, and a 5 mL plasma preparation tube (PPT) (tube E) was collected from each respondent who consented to the blood draw. While respondents were encouraged to fast from the night before, we also collected VBS from respondents who could not or did not fast.

### Standard operating procedure

The phlebotomists were trained in the study protocol before each phase of the study commenced. The research team explained the SOP in detail, including participant confidentiality, good clinical and laboratory practices, blood-draw technique, sequence of vacutainers, sample shipment in cold-chain, and sample processing. Each state team supervisor and the phlebotomist were provided with a checklist of required materials for sample collection (S1 Table). If the local laboratory was more than two hours from the collection site, an automated centrifuge machine was included to process the blood samples on-site.

During research initiation in each state, the research team supervised the first VBS collection to certify each phlebotomist. On the day of VBS collection, a unique barcode ID was assigned to each respondent and added to the sample acquisition form, the vacutainers, and the study database using a Computer-Assisted Personal Interview (CAPI) device. Assigning a unique barcode ID ensured accurate identification while maintaining confidentiality during subsequent sample processing steps. The trained staff followed good phlebotomy practices [29] during blood collection to prevent the likelihood of sample contamination and transmission of infectious disease agents [30]. A tourniquet was applied to the upper arm, proximal to the antecubital fossa, to help identify the median cubital vein [30]. The venipuncture area was sanitized using an alcohol swab. After letting the area air dry, a single-use two-way 22-gauge needle was inserted into the vein, and the specimen was collected using the vacuum extraction system of blood collection. The tourniquet was released as soon as the blood flow was visible in the first vacutainer [31]. The order of the vacutainers for sample collection is given in Table 1. The sequence of the tubes was decided to prevent cross-contamination of the additives between vacutainers [32]. In the event of a residual scar, pain, edema, abscess, cellulitis, adverse events in the past during VBS collection, or any other discomfort in both arms, VBS collection was not done. Moreover, in some rare cases where it was difficult to stabilize the vein, the blood flow was slow or an insufficient quantity was collected, a respondent-consented second attempt was made to collect the remaining blood sample.

**Table 1:** Time stamp quality check (QC) results.

Timestamp checks		Target duration (hours)	Observed duration (hh^a^ mm^b^)		
			Median	Interquartile range (IQR)	
Starting point	Ending point			Q1	Q3
Specimen collection time	Time of shipment to the local lab	4 hh	2 hh 47 mm	1 hh 20 mm	5 hh 30 mm
Time of shipment to the local lab	Receiving time at the local lab	4 hh	45 mm	28 mm	2 hh
Receiving time at the local lab	Time of shipment to the central lab	6 hh	2 hh 6 mm	1 hh 19 mm	3 hh 58 mm
Time of shipment to the central lab	Receiving time at the central lab	24 hh	20 hh	14 hh 39 mm	33 hh 9 mm

^a^Hours

^b^Minutes

As per the manufacturer’s instructions for use [33], the SSTs were gently inverted five times and kept vertical for 30 minutes at room temperature before shipment or on-site processing. It allowed the blood to clot and prevented the suspension of cellular components in the serum that could affect the analyte concentration [34]. The EDTA tubes and the PPTs were gently inverted 10 times to mix the blood with the additives [33]. After mixing, the tubes were immediately transferred to the cold chain box. As soon as VBS was collected from the first respondent, a single-use factory-calibrated Appresys temperature logger was placed with the samples to record the temperature of the cold chain at a logging interval of 5 minutes until it reached the central laboratory in Delhi. The details of VBS collection such as date, time, and number of tubes collected corresponding to each unique barcode, temperature logger ID, collection location, and shipment destination were entered in the sample acquisition form and the CAPI.

### Sample packaging, processing, shipment, and storage

All the vacutainers were placed vertically in a tube tray in an insulated Styrofoam box. Gel packs were added to maintain an ambient cold chain temperature of 2–8°C. Air-filled polyethylene packaging films were placed to stabilize the tubes and prevent their direct contact with the gel packs. The EDTA tubes were placed in the center and the SSTs and PPTs at the periphery to prevent whole blood from freezing. Within four hours of collection, the samples were shipped to a local Metropolis Healthcare laboratory for centrifugation or processed on-site if required.

The sample collection and receiving details were entered into the blood management system (BMS) by trained staff at the local laboratory. Each state had unique login credentials to record these details. Within two hours of reaching the local laboratory, SSTs and PPTs were centrifuged at 3500 revolutions per minute (rpm) for 10 minutes. The time of sample processing was entered and images of the centrifuged samples were taken and uploaded. The images were used to record and compare the hemolysis status of samples at the local laboratories and after they were received at the central laboratory in Delhi. The EDTA tubes were stored unprocessed in the cold chain. The samples were packed per the protocol described in the previous section and shipped to the central Metropolis Healthcare laboratory in Delhi within six hours of receiving samples at the local laboratory. The time of shipment was also entered into the BMS to track the samples in real time. VBS collection and fieldwork were completed in April 2024. Data from the BMS were downloaded and cleaned to generate a master file with sample collection and shipment information. The cleaned master data file was accessed to complete the analysis for this paper on July 23, 2024. Descriptive analysis was done to determine the median and interquartile range of shipment duration between different time points. The median sample shipment duration from the local laboratories to the central laboratory in Delhi was 20 hours (Table 1).

The central laboratory team registered the sample barcodes and entered the time of receipt on the BMS. The tubes A, B, and E were centrifuged again at 3500 rpm for 10 minutes, and serum-based and whole blood-based assays were run. All routine assays were performed within eight hours, and specialized tests within 24 hours of receiving the samples at the central laboratory. Table 2 lists the blood-based assays that were selected as potential risk factors for the pathophysiology of dementia. These biomarkers are related to inflammatory, metabolic, vascular, and neurodegenerative changes and could be the underlying modifiable risk factors for dementia and overall cognitive decline [35–39].

**Table 2:** List of blood-based assays conducted for each study participant.

Biospecimen	Biomarker	Instruments and assay methods
Serum-based assays (Central Metropolis laboratory)	**Lipid profile**	
	Total cholesterol	Roche Cobas 8000: Cholesterol Oxidase- Peroxidase
	High-density lipoprotein (HDL-c)	Roche Cobas 8000: Accelerator Selective Detergent
	Low-density lipoprotein (LDL-c)	Roche Cobas 8000: Homogeneous Enzymatic Colorimetric Assay
	Triglycerides	Roche Cobas 8000: Glycerol Phosphate Oxidase
	**Metabolic panel**	
	Bilirubin (total and direct)	Roche Cobas 8000: Diazotized Sulfanilic Acid (Modified Jendrassik & Grof)
	Creatinine	Roche Cobas 8000: Modified Jaffe
	Calcium	Roche Cobas 8000: NM – BAPTA
	Total protein	Roche Cobas 8000: Biuret
	Albumin	Roche Cobas 8000: Bromcresol Green (BCG)
	Alanine aminotransferase (ALT)	Roche Cobas 8000: NADH without P5P
	Aspartate aminotransferase (AST)	Roche Cobas 8000: NADH without P5P
	Alkaline phosphatase	Roche Cobas 8000: Para-Nitrophenyl-phosphate
	Gamma-glutamyl transferase (GGT)	Roche Cobas 8000: L-Gamma-glutamyl-3-carboxy−4–nitro analyte
	Blood urea nitrogen (BUN)	Roche Cobas 8000: Urease
	Homocysteine	Architect i2000: Chemiluminescence Microparticle Immunoassay (CMIA)
	Vitamin B12	Roche Cobas 8000: ECLIA Electrochemiluminescence Immunoassay
	25 Hydroxy Vitamin D	Roche Cobas 8000: Electrochemiluminescence Immunoassay (ECLIA)
	Folic acid	Roche Cobas 8000: Electrochemiluminescence Immunoassay (ECLIA)
	Ferritin Iron	Roche Cobas 8000: Electrochemiluminescence Immunoassay (ECLIA)
	Iron	Roche Cobas 8000: FerroZine
	Unsaturated iron binding capacity (TIBC)	Roche Cobas 8000: FerroZine
	Uric acid	Roche Cobas 8000: Uricase
	Thyroid function tests:	Roche Cobas 8000: Electrochemiluminescence Immunoassay (ECLIA)
	1. Total thyroxine (T4)	
	2. Total Triiodothyronine (T3)	
	3. Thyroid Stimulating Hormone (TSH: Ultrasensitive)	
	High-sensitivity C-reactive protein (hsCRP)	Atellica NEPH 630: Nephelometry
	N-terminal Pro-brain natriuretic peptide	Roche Cobas 8000: Electrochemiluminescence Immunoassay (ECLIA)
	Lipoprotein (a)	Roche Cobas 8000: Immunoturbidimetric Assay
Whole blood-based assays (Central Metropolis laboratory)	Complete blood cell count:	Beckman Coulter UniCel DxH 800: Electrical Impedance
	1. Red blood cell count	
	2. White blood cell count	
	3. Platelet count	
	Hemoglobin	Beckman Coulter UniCel DxH 800: Spectrophotometry
	Differential leucocyte count (DLC):	Beckman Coulter UniCel DxH 800: VCS (Volume, Conductivity and Scatter)
	1. Neutrophils	
	1. Basophils	
	2. Eosinophils	
	3. Lymphocytes	
	4. Monocytes	
	Glycosylated hemoglobin (HbA1c) levels	Tosoh G8: High-Performance Liquid Chromatography (HPLC)
Plasma-based assays (AIIMS, Delhi)	**Neurodegenerative biomarkers**	
	1. β-amyloid 42 (Aβ42 and)	Quanterix SIMOA-HD-X: Simoa^TM^ Neurology 4-Plex E (AB40, AB42, GFAP, NF-light) Reagent kit (Catalog- 103670)
	2. β-amyloid 40 (Aβ40) protein	
	3. Neurofilament light (NFL)	
	4. Glial fibrillary acidic protein (GFAP)	
	5. Total tau (tubulin-associated unit) protein	Simoa^TM^ Tau 2.0 Reagent kit (Catalog 101552)
	6. Phosphorylated tau^181^ (p-tau^181^) protein	Simoa^TM^ p-Tau 181 v2.1 Reagent kit (Catalog 104111)

The plasma, residual serum, and whole blood samples were separated into aliquots and stored in cryovials in −80°C freezers at Metropolis Healthcare Ltd, Delhi. Each aliquot was assigned a unique cryogenic barcode. Plasma cryovials were shipped to the Department of Biophysics, AIIMS, New Delhi for running AD-related biomarker assays, including β-amyloid 42, β-amyloid 40, tau proteins (total and phosphorylated tau^181^), neurofilament light (NFL), and glial fibrillary acidic protein (GFAP). These assays were run using an automated, ultrasensitive Single Molecule Array (Simoa) analyzer (Quanterix HD-X).

### Assay results and respondent health reports

The assay results from each specimen were reviewed and approved by the authorized signatory at Metropolis Healthcare laboratory, and the data were uploaded to the BMS system. The data were reviewed fortnightly by the biomarker experts of the research team. The research team linked unique barcodes to their corresponding respondent IDs to generate blood assay reports. These health reports were shared with the field research team in India. The field investigators returned these reports to each respondent by hand. The respondents with assay values beyond normal limits were advised to consult their physicians. Handing over the reports in person was aimed at maintaining a good rapport with the respondents, which also motivated other respondents to participate in blood draws, and hence contributed to a good response rate.

### Quality control

Prior research evidence confirms that maintaining good pre-analytical sample quality is crucial to obtaining reliable assay results [24,40]. This is particularly important for the biomarkers with lower concentrations in the blood and factors that negatively affect sample quality can make quantifying these biomarkers challenging [25]. In light of this, we implemented multiple quality control (QC) checks throughout the VBS collection process. First, the VBS collection was monitored to ensure that phlebotomists from each state followed the SOP. In case of unavailability of the designated phlebotomist, a new phlebotomist was trained virtually and monitored for the scheduled blood draw. Techniques, such as sanitizing the venipuncture area, removing the tourniquet within one minute [40], keeping the SSTs vertical at room temperature for 30 minutes before transportation/processing, and ensuring adequate mixing of samples with the anticoagulants by inverting the SSTs five times, and EDTA and PPTs ten times are important factors that help prevent sample contamination and hemolysis. Second, we utilized temperature loggers to record and monitor the temperature of each shipment until the samples were received at the central Metropolis Healthcare laboratory. Due to warm and humid weather in some states, it was challenging to maintain the cold chain with a relatively narrow permissible time window to conduct VBS collection and ship the samples to the local lab. To overcome this, the phlebotomists carried the gel packs in surplus. If the collection site was more than two hours away, especially in difficult-to-reach terrains, we limited the number of samples collected per day and kept additional gel packs under freezing temperatures in one of the participant’s refrigerators (if available and the family consented) or exchanged them from a primary/community health center in the vicinity. The gel packs were replaced before shipping the samples to the local labs. The logged data were analyzed fortnightly to monitor the cold chain temperature. The median receiving temperature of shipments was recorded to be 6.1°C (interquartile range: 4.1°C to 8.1°C) at the central Metropolis laboratory. Regulating the shipment temperatures within the target range is important to prevent changes in the levels of thermolabile analytes. A previous study from a national survey in India suggested that the levels of analytes, such as vitamin A, folate, and hemoglobin, drop with increasing temperature, while vitamin D, zinc, ferritin, and creatinine levels rise [41]. Vitamin B12, hsCRP, serum transferrin receptor, total and LDL cholesterol, HbA1c, glucose, and albumin, were found to be stable up to 24 hours at all temperatures (2° to >30°C) without centrifuging the whole blood specimens [41]. Plasma-based AD/ADRD biomarkers, such as p-tau^181^, NFL, GFAP, and Aβ42/Aβ40 ratio, show good stability when stored at +18°C for up to 24 hours [42–43]. Third, we recorded the time stamp for each respondent’s samples. The BMS auto-generated time stamp discrepancies helped notify the research team of sample shipment or processing delays. Correcting the impact of an increase in temperature and delays in sample processing [24] warrants temperature monitoring and real-time tracking of the samples. Fourth, all serum and whole blood-based assays were run at the central Metropolis lab to prevent measurement bias that could result from inter-equipment and inter-examiner variations [44]. As a pre-requisite to testing the samples, the central Metropolis Healthcare laboratory ran in-house QC samples daily as per NABL guidelines. As per protocol, if one of the QC samples was beyond three standard deviations (SD) from the laboratory-established mean or two QC samples were beyond two SD from the mean, the study samples were only tested after internal QC pass. We also independently monitored QC sample results on a real-time basis.

Additionally, we compared the hemolysis status of the samples at both the local and central laboratories. This step provided a check on sample handling and validated whether SOPs were followed at all steps. We utilized a subjective but standardized scale to inspect hemolysis in the samples [45]. To resolve any discrepancies in identifying sample hemolysis, a consensus decision was taken by the laboratory staff and the research team. We closely monitored and reviewed the hemolysis rate fortnightly. We collected blood samples from 3252 respondents. Out of these, samples from 184 SSTs (6.2%) and 37 PPTs (1.2%) were hemolyzed (overall hemolysis rate: 6.7%), ranging from mild to severe. Previous studies have reported a wide range of hemolysis rates (3.8% to 16.2%) in the collected venous blood samples [46–50]. These studies have also indicated that inadequate phlebotomist training and experience, longer tourniquet time, errors in sample collection technique, larger needle size, and improper sample handling are important factors contributing to sample hemolysis. The hemolysis rate in LASI-DAD indicated successful implementation and monitoring of the entire process, irrespective of the challenges faced. Our study demonstrates that it is feasible to collect VBS and ensure high-quality specimens in a nationally representative sample in India. The availability of high-quality VBS has allowed LASI-DAD to successfully measure AD/ADRD biomarkers that have relatively low blood concentrations, such as β-amyloid 42 and 40, phosphorylated tau-proteins, NfL, and GFAP. The stored study specimens may also provide additional research opportunities, should new novel assays become available in the future. Therefore, these VBS-based biomarker data may help to better understand the pathogenesis of AD/ADRD and offer potential dementia interventions in low- and middle-income countries.

### Improving VBS response rate and challenges

LASI-DAD is the first study in India to have conducted community-based VBS collection to quantify physiological and AD/ADRD-related biomarkers nationally. The overall response rate for VBS collection was 71.3%. Individual, community, and administrative factors for non-participation were observed based on informal feedback from the respondents, their family members, and field investigators.. The individual reasons observed included trypanophobia, unfavorable health conditions of some participants, and a prior experience of side effects following blood draws, such as hematoma, pain, swelling, and bruising. The community-driven refusals mainly arose from family members who were either not living with the respondent or reluctant to allow for any invasive procedures. Similar reasons for refusal to participate in community-based and clinical research have been reported in the past [51,52]. The field investigators addressed the concerns and created awareness through audio-visual aids on the safety of the VBS collection process. If the respondents were still unsure, the teams visited the respondents later to allow them to discuss it further with their physicians or family members. We also sought written permission from local and district or state-level health authorities whenever required. A final refusal was recorded if they did not consent to participate during the second visit. For the others, delayed receipt of administrative approvals from the local healthcare authorities due to unforeseeable reasons resulted in the conclusion of blood draws for a few sites.

Besides the refusals from respondents, high climate temperatures, and unfavorable road and terrain conditions, especially in the districts of Assam and Jammu and Kashmir (Kargil), were another challenge. These were overcome by using gel packs in surplus and replacing them frequently to prevent temperature rise.

## Conclusions

The methodology of VBS collection for LASI-DAD provides insights into developing and executing a systematic approach to collecting high-quality venous blood samples. It highlights the importance of rigorous training, monitoring compliance to SOPs, quality checks, and a prompt reporting system in implementing a successful VBS collection for a nationwide research study. Blood-based assays provide a cost-effective and minimally-invasive mass-screening way of objectively reflecting upon the overall health of older people in India. Additionally, the samples were assayed for biomarkers more specific to AD and ADRD. LASI-DAD encompasses data collection on a national level and provides an in-depth methodology for planning and implementation of VBS collection at the comfort of the participants without compromising on the quality of the samples. In conclusion, the methodology we developed over the course of the study is a promising tool that can serve as a model for future research involving community-based VBS collection to achieve assay results with high reliability and accuracy.

## Supporting information

S1 Table Equipment/Material checklist for venous blood specimen collection. (DOCX)
