# Supplementary material for: Methodology of community-based venous blood specimen collection for the harmonized diagnostic assessment of dementia for the Longitudinal Aging Study in India (LASI-DAD): Wave 2
Source: PLoS One. 2025 Jul 31;20(7):e0326917. doi: 10.1371/journal.pone.0326917 (PMC12312927; doi:10.1371/journal.pone.0326917)
Supplement: S1 Table — (DOCX) [file pone.0326917.s001.docx]

**S1 Table. Checklist of material and equipment required for venous blood specimen collection**

| Equipment/materials | Items |
| --- | --- |
| Personnel protective equipment and sanitizing material | Alcohol hand rub |
|  | Gloves |
|  | Face masks |
|  | Puncture-proof box |
|  | Biohazard bag |
| VBS collection kit (per respondent) | Two-way 22-gauge needle |
|  | BD^c^ Vacutainers (in the order of specimen collection) |
|  | 1. Tubes A and B: 3.5 mL Serum Separation Tube with gel separator (SST) |
|  | 1. Tube C: 2 mL EDTA tube |
|  | 1. Tube D: 3 mL EDTA tube |
|  | 1. Tube E: 5 mL Plasma Preparation Tube with gel separator (PPT) |
|  | Sterilized cotton |
|  | Alcohol wipes/swabs |
|  | Round adhesive bandage |
|  | Unique identification barcode |
| Shipping, processing, and packaging material | Insulated Styrofoam box |
|  | 8-10 gel packs for each Styrofoam box |
|  | Tube stands or trays |
|  | Air-filled polyethylene packaging film |
|  | Temperature loggers |
|  | Centrifuge machine^d^ |

^c^ Becton, Dickinson (BD) and Company, Franklin Lakes, New Jersey, USA

^d^ For locations that were more than two hours away from the local Metropolis laboratory, a portable centrifuge machine was carried to centrifuge the samples within 4 hours of collection.
